# Prognostic and Symptomatic Aspects of Rapid Eye Movement Sleep in a Mouse Model of Posttraumatic Stress Disorder

**DOI:** 10.3389/fnbeh.2013.00060

**Published:** 2013-05-31

**Authors:** Stephanie Anna Polta, Thomas Fenzl, Vladimira Jakubcakova, Mayumi Kimura, Alexander Yassouridis, Carsten Tobias Wotjak

**Affiliations:** ^1^Max Planck Institute of Psychiatry, Munich, Germany; ^2^Department of Pharmacology and Toxicology, Center for Chemistry and Biomedicine, University of Innsbruck, Innsbruck, Austria

**Keywords:** PTSD, animal model, biomarker, sleep fragmentation, REM sleep, vulnerability, fear conditioning, risk factor

## Abstract

Not every individual develops Posttraumatic Stress Disorder (PTSD) after the exposure to a potentially traumatic event. Therefore, the identification of pre-existing risk factors and early diagnostic biomarkers is of high medical relevance. However, no objective biomarker has yet progressed into clinical practice. Sleep disturbances represent commonly reported complaints in PTSD patients. In particular, changes in rapid eye movement sleep (REMS) properties are frequently observed in PTSD patients. Here, we examined in a mouse model of PTSD whether (1) mice developed REMS alterations after trauma and (2) whether REMS architecture before and/or shortly after trauma predicted the development of PTSD-like symptoms. We monitored sleep-wake behavior via combined electroencephalogram/electromyogram recordings immediately before (24 h pre), immediately after (0–48 h post) and 2 months after exposure to an electric foot shock in male C57BL/6N mice (*n* = 15). PTSD-like symptoms, including hyperarousal, contextual, and generalized fear, were assessed 1 month post-trauma. Shocked mice showed early onset and sustained elevation of REMS compared to non-shocked controls. In addition, REMS architecture before trauma was correlated with the intensity of acoustic startle responses, but not contextual fear, 1 month after trauma. Our data suggest REMS as prognostic (pre-trauma) and symptomatic (post-trauma) marker of PTSD-like symptoms in mice. Translated to the situation in humans, REMS may constitute a viable, objective, and non-invasive biomarker in PTSD and other trauma-related psychiatric disorders, which could guide pharmacological interventions in humans at high risk.

## Introduction

Posttraumatic Stress Disorder (PTSD) is an anxiety disorder that develops in 8–20% of individuals after having experienced a traumatic event involving extreme fear, helplessness, and horror (Kessler et al., 1995; Yehuda and LeDoux, 2007). The fact that not every individual develops PTSD after a traumatic experience suggests the existence of resilience and susceptibility factors describing a person’s ability to deal with the traumatic situation (Bush et al., 2007; Yehuda and Flory, 2007; Dudley et al., 2011). Pre-existing risk factors and biomarkers that would allow objective risk assessment might be of high medical relevance in occupational fields where traumatic events could be anticipated, such as emergency assistance and combat missions (Ramchand et al., 2010; Baker et al., 2012; McNally, 2012; Meyer et al., 2012). Susceptibility has been related to environmental conditions like a poor social network, lack of support, and severely stressful experiences in the past (Mealer et al., 2012; Meyer et al., 2012). Pre-trauma individual differences of predictive potential like hyperarousal (Pole et al., 2009), the efficiency in extinction learning (Lommen et al., 2013) and the existence of nightmares (van Liempt et al., 2013) have been observed. However, no objective biomarker has yet progressed into practical clinical use.

Sleep disturbances represent commonly reported complaints in PTSD patients, most frequently manifested in sleep-onset insomnia, sleep-maintenance insomnia, and nightmares (Harvey et al., 2003). Therefore, sleep disturbances have been considered a hallmark feature of PTSD (Ross et al., 1989; Germain, 2012), more than just a secondary symptom (Spoormaker and Montgomery, 2008), and have been proposed to contribute to the impaired fear extinction and fear extinction consolidation presented by PTSD patients (Levin and Nielsen, 2007; Germain et al., 2008; Spoormaker et al., 2010, 2012).

Polysomnographic studies point toward rapid eye movement sleep (REMS) abnormalities in PTSD patients (Mellman et al., 1995b, 2002; Breslau et al., 2004; Habukawa et al., 2007; Kobayashi et al., 2007; Insana et al., 2012). The nature of REMS changes appear to be complex and partially controversial. Reports include increased (Ross et al., 1994a,b; Engdahl et al., 2000) or decreased (Lavie et al., 1979; Glaubman et al., 1990) percentages of REMS, increased REMS density (Ross et al., 1994a,b, 1999; Mellman et al., 1995b, 1997), also related to the severity of PTSD (Mellman et al., 1995a), or no changes in the amount of REMS (Lavie et al., 1979; Dow et al., 1996; Mellman et al., 1997). However, recent studies indicated that REMS fragmentation (i.e., the appearance of short but frequent REMS episodes) seems to represent a major characteristic of disturbed sleep in PTSD patients (Insana et al., 2012), being related to the amount of sustained nightmares (Habukawa et al., 2007) and even being predictive of PTSD symptom severity (Mellman et al., 2002).

Sleep and particularly REMS plays a crucial role in facilitating memory of emotionally salient content (Wagner et al., 2001; Nishida et al., 2009; Popa et al., 2010). The amount of REMS during the REMS-rich second half of the night (Wagner et al., 2001; Groch et al., 2012) or during naps (Nishida et al., 2009) was associated with the enhancement of memory for emotional pictures. Emotional brain systems are selectively activated during REMS (Maquet et al., 1996; Braun, 1997). Also, REMS has been proposed to play a role in re-processing emotional information and stabilizing emotional reactivity (Horne, 2000; Wagner, 2002; Walker, 2009; Walker and van der Helm, 2009; Gujar et al., 2011; van der Helm et al., 2011; Baran et al., 2012; Groch et al., 2012). By the same token, REMS disturbances have been associated with increased emotional reactivity in humans (Rosales-Lagarde et al., 2012) and animals (Martinez-Gonzalez et al., 2004). Therefore, poor sleep (and especially REMS) may affect the emotional processing of traumatic experiences, and, thus, favor the development of PTSD.

The clinical diagnosis of PTSD requires evidence of long-lasting symptomatology for at least 1 month (American Psychiatry Association, 1994). Complaints of sleep impairments 1 month, but not 1 week, post-trauma have been shown to be significant predictors of PTSD symptom severity at 12 months post-trauma (Harvey and Bryant, 1998; Koren et al., 2002). Most importantly, sleep disturbances immediately prior to the traumatic experience have been shown to predict the development of subsequent psychiatric conditions (Bryant et al., 2010), and existing nightmares before deployment to Afghanistan predicted PTSD symptoms at 6 months post-deployment (van Liempt et al., 2013). Consequently, REMS architecture and nightmares were proposed as risk markers predicting the individual susceptibility for developing PTSD following a traumatic experience (Insana et al., 2012; van Liempt et al., 2013).

Given the relative ease of running longitudinal studies in animals but humans, animal models of PTSD might be particularly useful and instructive in demonstrating relationships between sleep architecture before and after trauma and PTSD severity (Pawlyk et al., 2008; Philbert et al., 2011). Therefore, we examined the acute and long-lasting effects of a traumatic event (electrical foot shock) on sleep in an established mouse model of PTSD (Siegmund and Wotjak, 2006, 2007a,b; Golub et al., 2009, 2011; Siegmund et al., 2009a,b; Dahlhoff et al., 2010; Pamplona et al., 2011; Herrmann et al., 2012; Sauerhöfer et al., 2012; Thoeringer et al., 2012). This model is based on exposure to a brief, intense electric foot shock. In consequence of the traumatic experience, mice develop hyperarousal, trauma-associated contextual fear and generalized fear persisting for at least 1 month. Behavioral changes coincided with a decrease in hippocampal volume (Golub et al., 2011). Chronic treatment with SSRIs (Siegmund and Wotjak, 2007a) or a CRH receptor 1 antagonist (Thoeringer et al., 2012) could ameliorate these symptoms. C57BL/6N mice appear to be particularly susceptible for the development of PTSD-like symptoms (Siegmund and Wotjak, 2007a; Siegmund et al., 2009a; Dahlhoff et al., 2010). Nevertheless, even inbred mice on this genetic background showed inter-individual differences in PTSD susceptibility (Siegmund and Wotjak, 2007a; Siegmund et al., 2009a). We could identify low hippocampal levels of *N*-acetyl aspartate (NAA) before trauma as a vulnerability marker (Siegmund et al., 2009b), but failed so in case of behavioral markers (Siegmund et al., 2009a). Therefore, we were interested to see whether REMS could serve by this means.

Our experiments were performed based on two hypotheses: (1) mice exposed to an electric foot shock develop long-lasting PTSD-like symptoms that coincide with alterations in sleep, in particular REMS. (2) REMS disturbances before and/or in the early aftermath of the shock have a predictive value in terms of severity of the PTSD-like phenotype, assessed 1 month after trauma.

## Materials and Methods

### Animals

Laboratory animal care and experimental procedures were in compliance with the European Union recommendations for the care and use of laboratory animals and all experimental procedures were approved by the Committee on Animal Health and Care of the State of Upper Bavaria (AZ55.2-1-54-2532-43-09).

Adult male mice (C57BL/6N, Martinsried, Germany; *n* = 16; 10–12 weeks of age at arrival) were used in this study. Animals were housed individually in customized home cages that also served as recording cages (26 cm × 26 cm × 35 cm, clear Lucide^®^ walls, wood shaves as bedding material) under inverse 12–12 h light-dark cycle (lights ON at 9 p.m.) with free access to food and water. Mice were randomly assigned to the experimental groups (non-shocked, *n* = 8; shocked, *n* = 8).

### Surgery

For sleep recordings, animals were implanted with electroencephalogram (EEG) and electromyogram (EMG) electrodes (gold wire with ball-shaped endings, soldered on standard PCB socket connectors). Surgery was performed under isoflurane anesthesia (Isofluran, DeltaSelect GmbH, Germany; anesthesia unit: AgnThoas AB, Sweden) with meloxicam as a perioperative analgesic (0.5 mg/kg body weight s.c., Metacam, Braun Melsungen, Germany). Mice were implanted with four epidural EEG electrodes (EEG 1, EEG 2, reference, ground) and bilateral EMG electrodes inserted into the nuchal musculature of the animals. Electrodes, fixation screws, and PCB socket were fixed to the skull with dental cement (Paladur, Heraeus-Kulzer, Germany). Post-operatively, meloxicam was added to the drinking water for 5 days (0.5 mg/kg body weight).

### Experimental design

**Figure 1:**
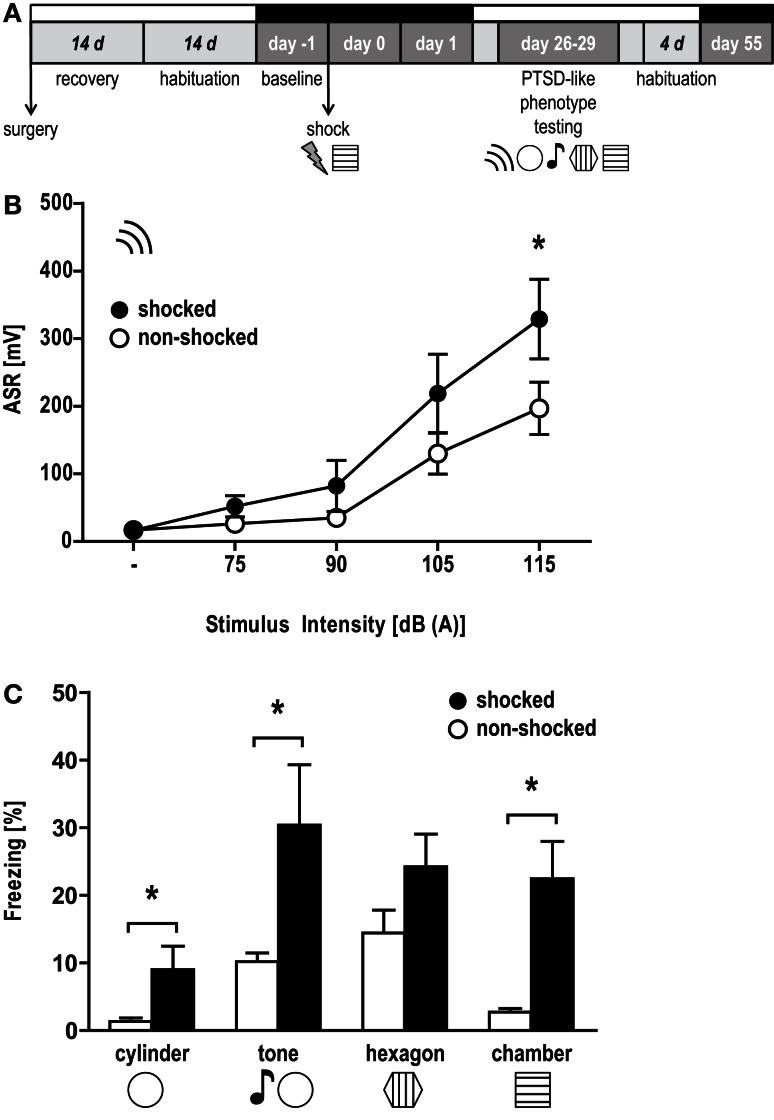
**Hyperarousal, contextual, and generalized fear in the aftermath of a trauma**. **(A)** Experimental design: mice were implanted with EEG and EMG electrodes (surgery), after which they were allowed to recover for 14 days. The subsequent habituation period to the recording cable-swivel system lasted for 14 days. EEG/EMG recordings are indicated by black bars above the respective days (baseline, day 0, day 1, day 55). On day 0, animals were exposed to two electric foot shocks (1.5 mA, 2 s) in the shock context. PTSD-like symptoms were assessed 1 month later (day 26–29) in the following order: (i) the acoustic startle response (ASR) and freezing behavior upon exposure to (ii) a completely new context (“cylinder”), (iii) a neutral tone in the cylinder (“tone”), (iv) a conditioning situation reminder context (“hexagon”), and (v) the original conditioning context (“chamber”). **(B)** ASR presented as group mean ± SEM based on 20 trials for each intensity presented in pseudorandom order. *p < 0.05 between shocked (black) vs. non-shocked control (white) group (unpaired *t*-test, one-tailed). **(C)** Percent time animals spent freezing (mean ± SEM). *p < 0.05 between shocked (black) vs. non-shocked (white) mice (unpaired*t*-test).

Figure 1A depicts the experimental schedule. After a recovery period from surgery of 2 weeks, mice were moved to the sound attenuated recording chambers [constant temperature (23 ± 1 °C), inverse light-dark cycle (12–12 h, lights ON at 9 p.m.)] where they were connected to the recording cable-swivel system which allows for free movement of the animals. Habituation to the system for 2 weeks was followed by EEG/EMG baseline recordings (day −1). On day 0, animals were exposed to two unsignaled electric foot shocks followed by two consecutive days of EEG/EMG recordings (day 0, day 1). After this recording period, animals were moved to the animal facility and remained there under the same housing conditions until behavioral testing 1 month after shock (day 26–29). Another month later, after habituation to the recording cable-swivel system for 4 days, EEG/EMG recordings were performed for additional 24 h (day 55).

### Shock procedure and PTSD-like symptoms

The setup has been described and displayed in detail before (Kamprath and Wotjak, 2004; Siegmund and Wotjak, 2007a). Shock presentations and behavioral tests were performed during the active diurnal phase between 9:30 a.m. and 4 p.m.

On day 0, animals of the shocked group were placed in a cubic conditioning chamber with a floor metal grid that was cleaned with water containing isoamylacetate (1:2000; banana aroma) between exposures. After an exploration period of 5 min, two scrambled electric foot shocks (1.5 mA, 2 s, 1 min interval between shocks) were delivered via the grid. One minute after delivery of the second shock, animals were returned to their home cages. Animals of the non-shocked group were only introduced to the conditioning chamber for an equivalent time of 7 min. The two experimental groups were spatially separated for the entire duration of the experiment to exclude possible influence of vocalization and olfactory signals.

One month after the shock, mice were tested for the presence of a PTSD-like phenotype based on measures of (1) acoustic startle response (ASR, day 26), (2) fear behavior in a neutral context (“cylinder,” day 28), (3) fear behavior in response to a sinusoidal sound in the cylinder (“tone,” day 28), (4) fear behavior in a context that contained reminder features of the conditioning context (“hexagon,” day 29), and (5) fear behavior in the original shock context (“chamber,” day 29). The order of testing was not randomized deliberately, but we followed the order described in our recent studies (e.g., Golub et al., 2009, 2011): first, we measured ASRs in a room different from the conditioning room. This allowed us to assess non-associative consequences of the foot shock. The order of context exposures (neutral context – reminder context – shock context) enabled direct assessment of context generalization, which would be influenced by prior recall of contextual fear memory (Wiltgen and Silva, 2007).

#### Hyperarousal (ASR)

The procedure and instrumentation has been described in detail elsewhere (Golub et al., 2009). In brief, mice were placed into a non-restrictive Plexiglas cylinder (inner diameter 4 cm, length 8 cm) within a sound-attenuated chamber (SR-LAB, San Diego Instruments SDI, CA, USA). Four different startle stimuli consisting of white noise bursts of 20 ms duration at 75, 90, 105, and 115 dB(A) intensity were presented against a constant background noise of 50 dB(A). During control trials, only background noise was present. The averaged inter-stimulus interval was 15 s (13–17 s, pseudo-randomized). After acclimation for 5 min, 10 control trials and 20 startle stimuli of each intensity were presented in a pseudorandom order in each test session. The startle amplitude was defined as the peak voltage output within the first 50 ms after stimulus onset. Plexiglas cylinders were cleaned with soapy water after each session.

#### Contextual and generalized fear

In order to assess fear generalization, mice were exposed to a neutral context (“cylinder,” Plexiglas, wood shavings, 1% acetic acid as an olfactory cue), followed by a neutral tone (80 dB, 9 kHz), and a context that contained a key feature of the original shock context (“hexagon,” non-transparent Plexiglas with metal grid floor as a dominant reminder of the shock context, 70% ethanol as an olfactory cue), each for 3 min. Contextual fear was assessed by exposing the animals to the original shock context (“chamber”) for 3 min (Golub et al., 2009). The tests for hyperarousal and generalized fear were performed in two completely different set-ups localized in two different labs. All tests were videotaped and freezing behavior (immobility except for respiration movements; Kamprath and Wotjak, 2004) over the entire 3 min intervals was quantified off-line by a trained observer blind to the experimental condition using the EVENTLOG scoring program (Robert Henderson, 1986).

### EEG/EMG recordings and analysis

The setup and recording techniques have been described in detail before (Fenzl et al., 2007, 2011; Jakubcakova et al., 2011). In brief, EEG and EMG signals were processed through a pre-amplifier (1000×, custom made) and a main amplifier (10×, custom made). The EEG signals were analog band-pass filtered (0.5–29 Hz, filter frequency roll off 48 dB/octave) and digitized at a sampling rate of 64 Hz (AD board, NI PCI-6070, National Instruments, Austin, TX, USA). Root mean square was applied to the non-filtered EMG signals before digital conversion (sampling rate: 64 Hz). Fast Fourier transform (FFT) was applied on consecutive 4 s intervals of the EEG data for spectral analysis. Vigilance states wakefulness (WAKE), non-rapid eye movement sleep (NREMS), and REMS were classified semi-automatically using a FFT-algorithm spectral analysis on a LabVIEW ^®^-based acquisition program (SEA, Cologne, Germany). The frequency bands considered in the study were: δ (0.5–5 Hz), θ (6–9 Hz), α (10–15 Hz), η (16–22.5 Hz), and β (23–31.75 Hz). The applied scoring algorithm was adapted from a previous report originally based on experiments in rats (Louis et al., 2004). The delta band and theta band were additionally specified (δ = δ × α/η × β and θ = θ × θ/δ × α; Fenzl et al., 2007). Based on the changes in the EEG signals, sleep stages were defined as follows: (a) if EMG-amplitude was above a manually set threshold (separating movement from no movement) the stage was defined as WAKE. WAKE episodes that lasted less than three epochs (12 s), were defined as microarousals (MA) (Léna et al., 2004). (b) If the calculated δ-value was below a manually set threshold (separating high delta power from low delta power) and (c) the calculated θ-value was above a manually set threshold (separating high theta power from low theta power) the stage was defined as REMS. All other sleep stages were defined as NREMS. All semi-automatically scored sleep stages were proof-read manually. One animal of the non-shocked group was excluded from the sleep-wake analysis due to poor quality of the EEG signals. Twenty-four hour recordings of day −1 (baseline), day 0 (shock day), day 1 (1 day after shock), and day 55 (2 months after shock) were scored by a sleep assessment expert who was unaware of the experimental condition of the animals.

Sleep composition and architecture were scrutinized by assessing the time spent in MA, WAKE, NREMS, and REMS, the number of these events, their mean durations and the number of transitions between them. Additionally, the EEG power of defined frequency bands (delta, theta, alpha, eta, beta) within WAKE, NREMS, and REMS was computed by measurements of the area under the curve (AUC) using the trapezoid rule (technique for approximating the definite integral by approximating the region under the graph of the function as a trapezoid and calculating its area). All sleep parameter data were normalized to the group mean value under baseline conditions and are shown as change to baseline (with 100% denoting baseline levels in the experimental groups).

**Figure 2:**
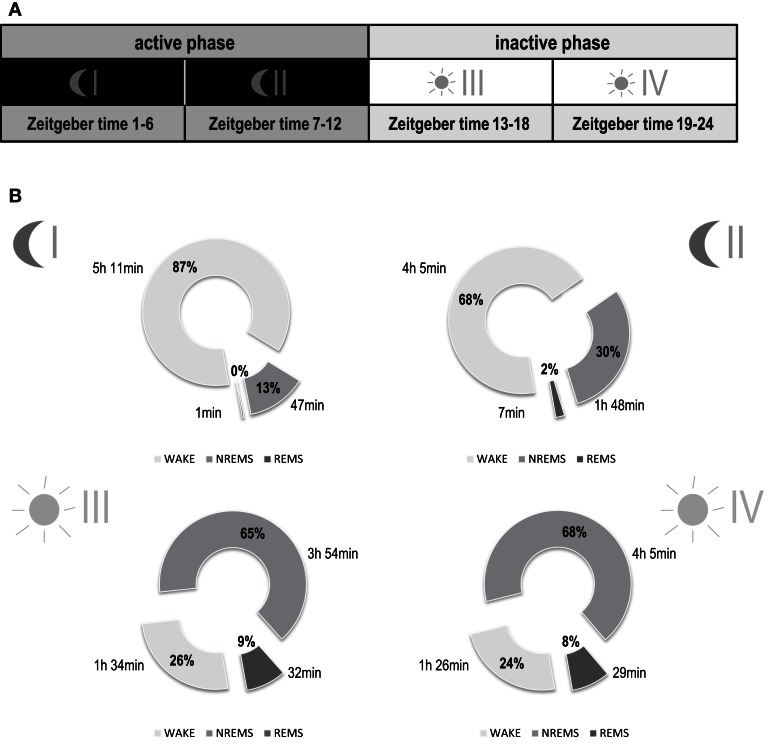
**Basal distribution of vigilance states of mice under reverse light-dark conditions**. **(A)** Illustration of sub-division of total recording time into four separate time intervals: phase I (first half of the dark period: Zeitgeber time 1–6 h), phase II (second half of the dark period: Zeitgeber time 7–12 h), phase III (first half of the light period: Zeitgeber time 13–18 h), and phase IV (second half of the light period: Zeitgeber time 19–24 h). **(B)** Sleep-wake behavior before trauma. Data are represented as means including both experimental groups (*n* = 15). Parts of the circle diagram show percentage of time mice spent in wakefulness (WAKE, light gray), non-rapid eye movement sleep (NREMS, gray), and rapid eye movement sleep (REMS, dark gray). Each diagram shows a 6-h phase of the circadian rhythm as illustrated in **(A)**. No differences were observed in baseline vigilance states when comparing the two experimental groups (shocked vs. non-shocked, 2-way ANOVA). Phase I [Mean ± SEM (%)]: WAKE, non-shocked: 83.0 ± 3.5, shocked: 89.6 ± 2.8; NREMS: non-shocked: 16.4 ± 3.4, shocked: 10.3 ± 2.8; REMS: non-shocked: 0.6 ± 0.2, shocked: 0.2 ± 0.1. Phase II [Mean ± SEM (%)]: WAKE, non-shocked: 67.8 ± 1.2, shocked: 68.4 ± 4.1; NREMS: non-shocked: 30.0 ± 1.0, shocked: 30.0 ± 3.8; REMS: non-shocked: 2.3 ± 0.3, shocked: 1.6 ± 0.4. Phase III [Mean ± SEM (%)]: WAKE, non-shocked: 28.0 ± 1.7, shocked: 24.5 ± 1.3; NREMS: non-shocked: 63.6 ± 1.3, shocked: 66.4 ± 1.1; REMS: non-shocked: 8.5 ± 0.6, shocked: 9.1 ± 0.6. Phase IV [Mean ± SEM (%)]: WAKE, non-shocked: 22.2 ± 1.3, shocked: 24.1 ± 1.2; NREMS: non-shocked: 65.9 ± 1.6, shocked: 65.5 ± 1.0; REMS: non-shocked: 8.9 ± 0.8, shocked: 7.6 ± 0.5.

Statistical evaluations and graphical presentation refer to the analysis of four separate time intervals (Figure 2A): phase I (first half of the dark period: Zeitgeber time 1–6 h), phase II (second half of the dark period: Zeitgeber time 7–12 h), phase III (first half of the light period: Zeitgeber time 13–18 h), and phase IV (second half of the light period: Zeitgeber time 19–24 h). The analysis of the sleep-wake behavior was based on 6 h intervals since differences in the development of WAKE, NREMS, and REMS amounts can be observed over the 24 h light-dark-rhythm, also in humans. Because animals underwent fear conditioning during phase I on day 0, this period was excluded from the analysis.

### Statistical analysis

Nominal level of significance was pre-set at *p* < 0.05. Effects of group and stimulus intensity on the ASR were statistically analyzed by analysis of variance (ANOVA) for repeated measurements with the experimental group as a between-subjects and stimulus intensity as a within-subjects factors. Group effects on the freezing behavior were statistically analyzed by *t*-tests for independent samples. Group, day and group × day interaction effects on the sleep-wake architecture variables were statistically analyzed by multivariate analyses of variance (MANOVAs) with repeated measures designs separately for each phase. To avoid possible collinearities evoked by the multitude of the considered sleep variables, the sleep parameters were divided into subgroups of dependent variables for which MANOVA was performed. In case of significant factor effects (main and/or interaction effects), univariate *F*-tests followed, identifying those variables on which these effects were significant. Subsequently, *post hoc* tests (contrast tests in MANOVA) were applied on the variables showing significant factor effects in order to investigate the significance of differences between groups and days. To keep the type I error less or equal to 0.05, all *a posteriori* tests (univariate *F*-tests, contrast test in MANOVA) were performed with the Bonferroni correction. Associations between behavioral data and sleep parameters were tested for significance by means of the Pearson and Spearman correlation without correction for multiple comparisons.

## Results

### Trauma exposure induces PTSD-like symptomatology

Acoustic startle response measures (Figure 1B) revealed a trend toward elevated responses depending on stimulus intensities [stimulus × shock, *F*(1, 4) = 2.09, *p* = 0.09] and a marginally significant effect of shock on the startle response of the animals [*F*(1, 1) = 3.02, *p* = 0.10]. On the basis of our previous studies (Golub et al., 2009, 2011; Herrmann et al., 2012; Sauerhöfer et al., 2012) we expected the shocked mice to show increased ASRs, becoming evident in particular in response to white noise burst of 115 dB intensity. Therefore, we additionally compared the startle responses of shocked and non-shocked mice at 115 dB. Accordingly, the startle response of shocked mice was significantly increased [*t*(14) = 1.88, *p* < 0.05, one-tailed unpaired *t*-test]. Furthermore, shocked animals displayed increased freezing during exposure to the conditioning context [“chamber”: *t*(14) = 3.542, *p* < 0.005], the novel context [“cylinder”: *t*(14) = 2.175, *p* < 0.05] and to a neutral “tone” [*t*(14) = 2.239, *p* < 0.05], but not to the context resembling the conditioning situation [“hexagon”: *t*(14) = 1.66, *p* = 0.12] (Figure 1C). Together, these data indicate that shocked mice maintained high levels of arousal, contextual fear, and context generalization 1 month after the trauma.

### Baseline sleep-wake behavior follows a normal day-night rhythm

For each recording day, continuous 24 h recordings were divided into four phases of 6 h each, active (dark) phases I and II and inactive (light) phases III and IV respectively (Figure 2A). Under baseline conditions, all animals displayed a normal sleep-wake pattern over the dark-light cycle with higher amounts of wakefulness during the active phase (dark phase; 77.3 ± 13.0%) and higher amounts of NREMS (65.4 ± 0.4%) and REMS (8.5 ± 0.4%) during the inactive phase (light phase) (Figure 2B; for basal levels reported separately per group, see Figure Legend). No differences for any of the vigilance states were observed between experimental groups before trauma (MANOVA, *p* > 0.05).

### Shock exposure leads to sustained increase in REMS

**Figure 3:**
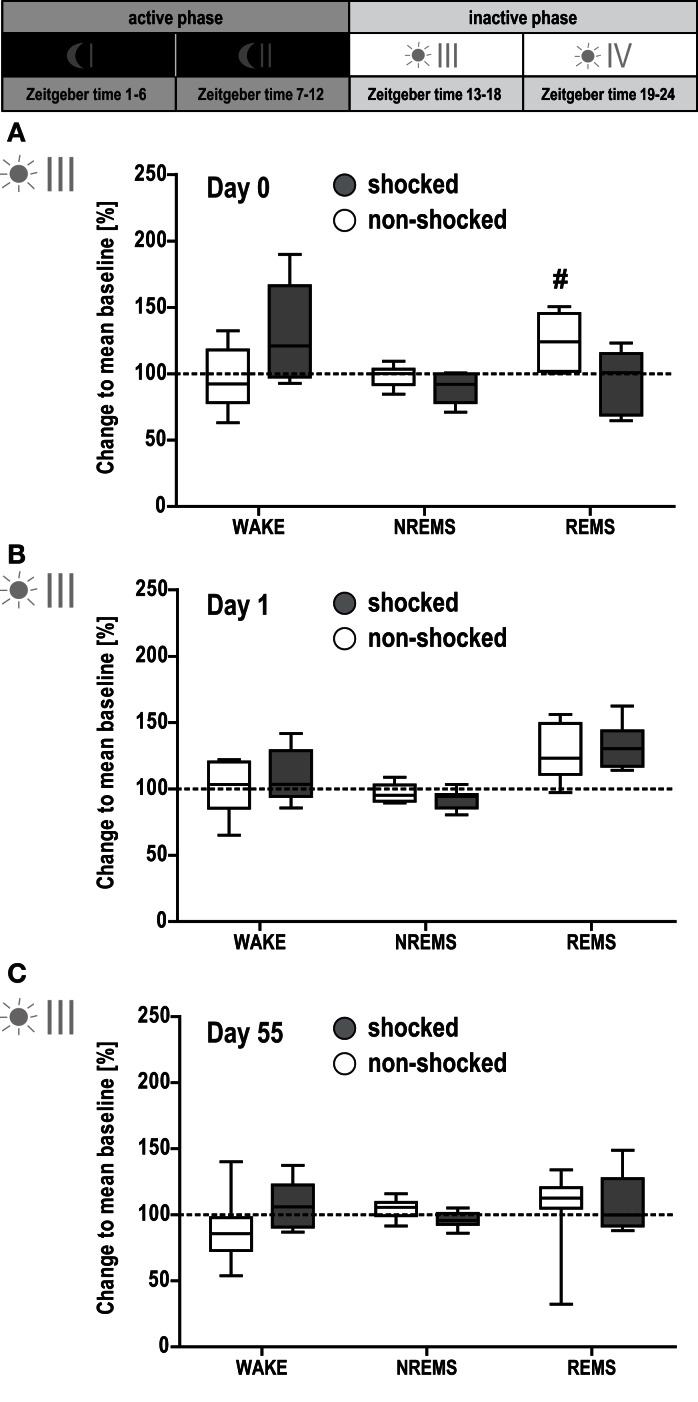
**Effect of shock on vigilance states during the first half of the inactive phase**. Sleep-wake behavior of the shocked and non-shocked mice was recorded on the day of the shock [day 0, **(A)**], the day after the shock [day 1, **(B)**] and 2 months after the shock [day 55, **(C)**; for experimental schedule, see Figure 1A]. For each recording day, results of the first half of the inactive (i.e., light) phase are depicted. The individual graphs show the change in time animals spent awake (WAKE), in non-rapid eye movement sleep (NREMS) or rapid eye movement sleep (REMS) relative to the group mean under baseline conditions (dotted horizontal line). Multivariate analysis revealed a significant group × day interaction effect on the conditioning day [day 0, A; group × day: *F*(9, 90) = 2.78, *p* < 0.01; day: *F*(9, 90) = 6.57, *p* < 0.001]. This effect could be localized for REMS [group × day: *F*(3, 39) = 3.90, *p* < 0.02]. Controls showed an increased amount of REMS during phase III on day 0, i.e., after their exposure to the novel context [day 0 vs. baseline: *F*(1, 6) = 17.95, *p* < 0.008]. However, the comparison between groups did not reveal a statistically significant difference (*p* < 0.05). Data are presented as box and whisker plots with boxes showing lower quartile, median and upper quartile, and whiskers showing the minimum and maximum sample. #*p* < 0.008 Indicates a statistically significant difference to baseline condition (*post hoc* contrast tests in MANOVA; Bonferroni corrected).

In order to visualize changes in sleep architecture due to shock exposure, all data were normalized to the group means obtained under baseline conditions and expressed as percent change to the respective baseline value. The magnitude of change was compared to that of non-shocked controls. Shocked and non-shocked animals showed a similar circadian distribution of time spent in WAKE, MA, NREMS, and REMS during their active phases I and II on all experimental days post-trauma (MANOVA, *p* > 0.5, data not shown). Additionally, there were no significant group differences in any sleep-wake parameter during the first part of the inactive phase (phase III; Figure 3).

**Figure 4:**
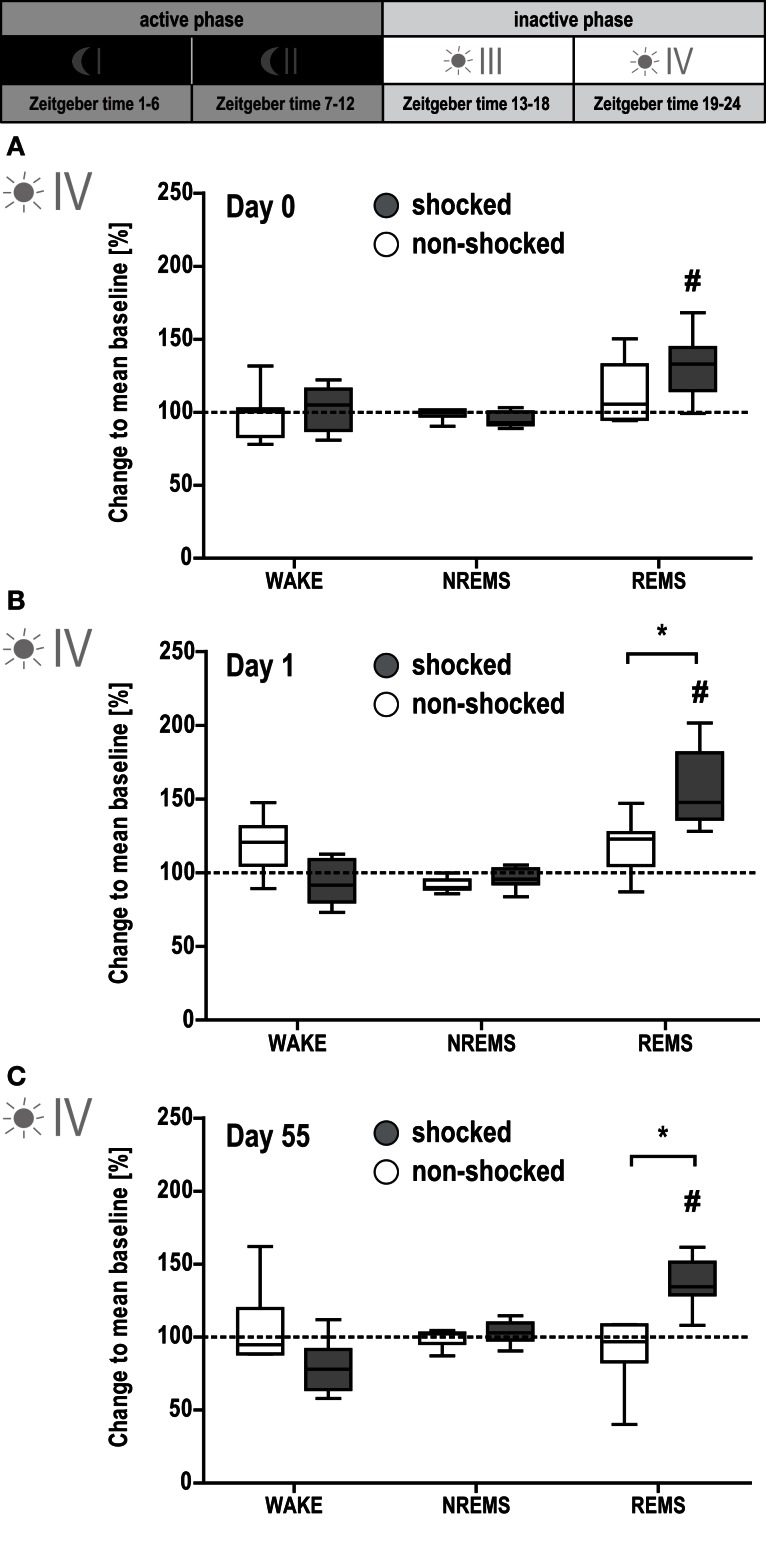
**Shocked mice show long-lasting increase in REMS**. Sleep-wake behavior of the mice was recorded on the day of the shock [day 0, **(A)**], the day after the shock [day 1, **(B)**], and 2 months after the shock [day 55, **(C)**; for experimental schedule, see Figure 1A]. For each recording day, results of the second half of the inactive (i.e., light) phase are depicted. The individual graphs show the change in time animals spent awake (WAKE), in non-rapid eye movement sleep (NREMS) or rapid eye movement sleep (REMS) relative to the group mean under baseline conditions (dotted horizontal line). Data are presented as box and whisker plots with boxes showing lower quartile, median and upper quartile, and whiskers showing the minimum and maximum sample. *p < 0.0125 indicates a statistically significant difference between shocked (gray) vs. non-shocked control (white) group (*post hoc* contrast tests in MANOVA; Bonferroni corrected). #*p* < 0.008 indicates a statistically significant difference to baseline condition (*post hoc* contrast tests in MANOVA; Bonferroni corrected).

However, MANOVA for the second part of the inactive phase (phase IV) revealed significant group [*F*(2, 11) = 7.81, *p* < 0.01] and day effects [*F*(8, 90) = 6.51, *p* < 0.001], and group × day interaction [*F*(8, 90) = 3.29, *p* < 0.01]. Follow-up univariate testing showed the significant effects being associated with REMS [group: *F*(1, 13) = 9.21, *p* < 0.01; day: *F*(3, 39) = 13.41, *p* < 0.01; group × day: *F*(3, 39) = 5.53, *p* < 0.001]. Shocked mice displayed increased REMS levels after the shock [day 0 vs. baseline: *F*(1, 7) = 16.06, *p* < 0.005; Figure 4A]. This elevation was maintained on day 1 [day 1 vs. baseline: *F*(1, 7) = 58.92, *p* < 0.001], reaching significance compared to non-shocked controls [shocked vs. non-shocked: *F*(1, 13) = 9.30, *p* < 0.01; Figure 4B]. Importantly, even 2 months after the traumatic event, REMS levels remained increased during this phase [day 55 vs. baseline: *F*(1, 7) = 33.63, *p* < 0.001; shocked vs. non-shocked: *F*(1, 13) = 18.73, *p* < 0.001; Figure 4C].

Since animals with a history of shock experience showed sustained elevation of time spent in REMS, we further evaluated the nature of this increase by a detailed analysis of the sleep-wake architecture as described previously (Fenzl et al., 2011; Jakubcakova et al., 2011). In this context, we assessed length and number of WAKE, MA, NREMS, and REMS episodes and the number of transitions between these vigilance states. Additionally, REMS quality (as measured by the EEG power in pre-defined frequency bands) was evaluated. We did not observe any significant group effect or factorial interaction for the active phases I and II or the inactive phases III and IV for any of these parameters (MANOVA, *p* > 0.05). Moreover, the increase in REMS early as well as 2 months after the trauma in shocked mice could not be explained by one single process: while some of the shocked animals showed an increase in the number of REMS episodes, others displayed an increase in the mean duration of the REMS episodes. In addition, shocked and non-shocked animals showed the same amount of sleep fragmentation in general and of REMS fragmentation in particular. Likewise, EEG power during REMS in pre-defined frequency bands was not affected by the traumatic event.

### REMS architecture before trauma is predictive of trauma-induced hyperarousal

**Figure 5:**
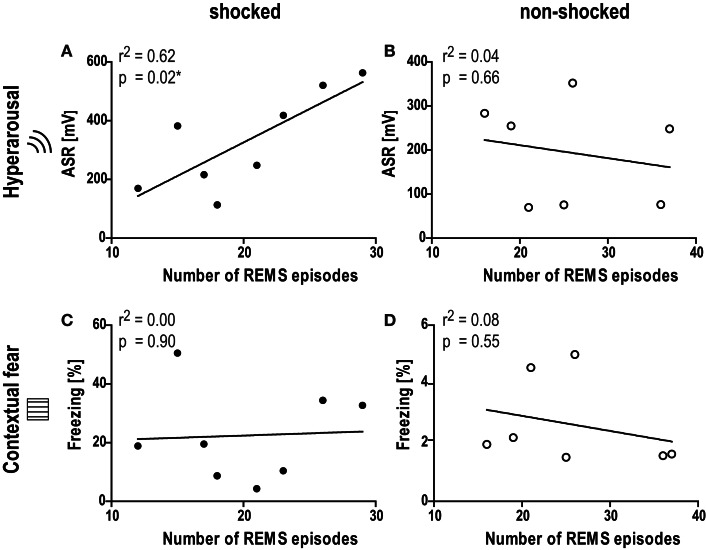
**Number of REMS episodes under baseline conditions is associated with the level of hyperarousal 1 month after the trauma**. Graphs show the correlation between the number of REMS episodes under baseline conditions in phase IV (day −1, Zeitgeber time 18–24) and **(A,B)** the acoustic startle response at intensity of 115 dB (ASR, day 26), or **(C,D)** the freezing behavior in the shock context (chamber, day 29) 1 month after the shock. Black circles: shocked group, white circles: non-shocked control group. *r*^2^ Correlation coefficient as obtained from linear regression analysis (Pearson correlation).

According to our hypothesis that REMS disturbances before and/or in the early aftermath of the shock could have a predictive value in terms of severity of the PTSD-like phenotype, correlation analyses were performed between behavioral data (startle, freezing to the context) and various REMS parameters (REM%, number of REM episodes, mean duration of REMS episodes, transitions from and to REMS) obtained on day −1 (baseline) and day 0 (immediately after shock). The number of REMS episodes during the second half of the inactive phase (phase IV) before trauma (baseline; day −1) was significantly correlated with the intensity of the startle response evoked by acoustic stimuli of 115 dB 1 month after trauma (day 26; Figure 5A; Pearson correlation coefficient *r* = 0.78; Spearman correlation coefficient *r* = 0.76; *p* < 0.05). Equally strong correlations were observed between the ASR and the transitions from (i) NREMS to REMS (Pearson *r* = 0.80, Spearman *r* = 0.76), (ii) REMS to WAKE (Pearson *r* = 0.72, Spearman *r* = 0.76), and (iii) REMS to NREMS (Pearson *r* = 0.80, Spearman *r* = 0.79, all *p* < 0.05), but not the total amount of REMS (Pearson *r* = 0.59, Spearman *r* = 0.62, *p* > 0.05). Therefore, low REMS continuity but not REMS duration was related to hyperarousal severity. By applying a linear fit on the association between REMS episodes and ASR, we found that more than 60% of the ASR variance could be explained by the above-described REMS continuity parameters. Non-shocked mice did not show any of such correlations (Figure 5B; Pearson *r* < 0.22, Spearman *r* < 0.44, *p* > 0.35). Additionally, none of the parameters showed significant associations with the freezing response in the shock context in both groups (Figures 5C,D; shocked: Pearson *r* < 0.21, Spearman *r* < 0.17, *p* > 0.62; non-shocked: Pearson *r* < 0.38, Spearman *r* < 0.32, *p* > 0.50). Taken together, these results indicate that the measure of REMS continuity under baseline conditions is associated with the degree of hyperarousal emerging after the exposure to a traumatic event.

## Discussion

The diagnostic criteria for PTSD consist of the persistence of three major symptom clusters for at least 1 month: re-experiencing (flashbacks), avoidance, and hyperarousal (American Psychiatry Association, 1994). Sleep, and REMS disturbances in particular, were suggested as a hallmark of PTSD almost 25 years ago (Ross et al., 1989). In addition, they have been proposed as possible pre-trauma indicators of the individual susceptibility for developing PTSD (Mellman and Hipolito, 2006; Bryant et al., 2010; Insana et al., 2012; van Liempt et al., 2013). While it is difficult to adequately model the whole complex etiology and symptomatology of human psychiatric disorders, modeling of selected phenotypes that reflect crucial features of the disease in animals provides a reliable tool to examine neurobiological correlates, mechanisms, and possible treatments (Siegmund and Wotjak, 2006). Here we employed an established animal model of PTSD, where mice are exposed to intense foot shocks, and subsequently develop long-lasting PTSD-like symptoms (Siegmund and Wotjak, 2007a; Golub et al., 2009, 2011; Pamplona et al., 2011). These symptoms include contextual as well as generalized fear (“avoidance symptoms”), increased startle response (“hyperarousal symptoms”) and, as shown in the present study, an elevated amount of REMS that lasts for more than 2 months after the trauma. In addition, low levels of REMS continuity before the trauma (i.e., under baseline conditions) were correlated with the intensity of ASRs (i.e., hyperarousal), but not contextual fear, 1 month after the trauma.

Various clinical studies provide converging evidence for REMS abnormalities in PTSD patients (Mellman et al., 1995b, 2002; Breslau et al., 2004; Habukawa et al., 2007; Kobayashi et al., 2007; Insana et al., 2012). Even though there are inconsistent reports about the nature of those changes, many studies point toward a disruption of REMS continuity in PTSD patients, as evident from shorter and more frequent REMS periods, increased REMS density, and more REMS to WAKE and REMS-to-stage-1 NREMS transitions (Ross et al., 1994a,b; Mellman et al., 1995b, 1997; Breslau et al., 2004; Kobayashi et al., 2007; Insana et al., 2012). REMS disturbances were described as being related to the amount of sustained nightmares (Habukawa et al., 2007) and even being predictive of PTSD symptom severity (Mellman et al., 2002).

Here we report that shocked animals showed an early increase in REMS amounts immediately after the trauma, which lasted for at least 2 months. These REMS alterations resemble human PTSD symptomatology where REMS alterations have been described as early symptoms in the aftermath of a traumatic event (Mellman et al., 2002, 2007; Mellman and Hipolito, 2006). Additionally, sleep deprivation early after trauma has been shown to reduce behavioral, physiological, molecular, and morphological responses to the trauma in a rat model of PTSD (Cohen et al., 2012).

There are numerous reports about acute effects of fear conditioning and other stressful events on sleep patterns in rodents (for a review, see Palma et al., 2000; Vazquez-Palacios and Velazquez-Moctezuma, 2000; Sanford et al., 2001, 2003a,b,c; Liu et al., 2003, 2009, 2011; Jha et al., 2005; Pawlyk et al., 2005, 2008; Madan et al., 2008; Wellman et al., 2008; Yang et al., 2009; Deschaux et al., 2010; Dasilva et al., 2011; Philbert et al., 2011). These studies differ substantially in crucial parameters such as the fear conditioning type, strength and frequency of conditioning, circadian time at conditioning, species, strain and gender of the animals, and their ambient light-dark-rhythm. Therefore, it is difficult to generate a comprehensive picture of their reported outcome. One common finding is that fear conditioning performed in the beginning of the inactive phase of the animals resulted in an acute (4–22 h after shock) decrease in REMS in rats (Palma et al., 2000; Sanford et al., 2001; Jha et al., 2005; Pawlyk et al., 2005; Liu et al., 2009, 2011; Yang et al., 2009; Deschaux et al., 2010) and mice (Liu et al., 2003; Sanford et al., 2003a,b,c; Wellman et al., 2008). However, a more detailed analysis of REMS architecture revealed a shock-related increase of sequential REMS episodes (interval between REMS episodes <3 min; Madan et al., 2008; DaSilva et al., 2011; Dasilva et al., 2011). Additionally, conditioning at the end of the active phase led to increased REMS in rats (Vazquez-Palacios and Velazquez-Moctezuma, 2000). Long-term effects of up to 21 days after trauma have been reported (Philbert et al., 2011) where a general increase in sleep fragmentation has been observed, without further differentiation of this effect between NREMS and REMS.

To the best of our knowledge, the present study is the first to show early onset, yet long-lasting changes in sleep architecture in terms of a sustained increase in REMS. We could not consistently observe fragmented REMS, possibly because of the *a priori* polyphasic sleep pattern in rodents (Campbell and Tobler, 1984; Welsh et al., 1986; Lo and Chou, 2004).

Analysis of REMS architecture before trauma revealed a significant association between the numbers of REMS episodes and the intensity of ASRs 1 month later. Similar relationships were observed for the transitions from NREMS to REMS, REMS to WAKE, and REMS to NREMS, but not for total REMS duration. Only animals confronted with the trauma, but not non-shocked controls, showed such a relationship between baseline REMS architecture and emerging hyperarousal symptoms. The findings indicate that low REMS continuity could serve as a risk marker predicting the individual susceptibility for developing PTSD-like hyperarousal in the aftermath of the trauma. It is of note that there was no similar association between pre-trauma REMS continuity and post-trauma contextual fear. This is in line with the diagnostic criteria of PTSD which clearly differentiate between non-associative hyperarousal symptoms on the one and associative memory intrusions and avoidance symptoms on the other hand, whereby sleep disturbances (including REMS fragmentation but not nightmares) and hyperarousal (including an increased startle response) belong to the same cluster (American Psychiatry Association, 1994). In addition, the lack of association corroborates our previous observation that hyperarousal and contextual fear represent distinct and dissociable consequences of traumatic experiences in mice (Siegmund and Wotjak, 2007b; Golub et al., 2009; Sauerhöfer et al., 2012).

At the current stage, we can only speculate about the neurochemical basis of the *a priori* differences in REMS continuity under baseline conditions and their relationship to the emergence of hyperarousal in the aftermath of the trauma. REMS as well as arousal depend on the intricate and orchestrated interaction of various neurotransmitter systems (Foote, 1980; Steriade and McCarley, 2005; Fort et al., 2009; Luppi et al., 2012). For instance, it has been suggested that disrupted REMS continuity in PTSD patients is caused by an increased noradrenergic (NA) tone (Southwick et al., 1999). Indeed, preclinical and clinical studies have implicated altered NA activity in contributing to hyperarousal and exaggerated amygdala reactivity, and increased NA levels have been found in the cerebrospinal fluid of PTSD patients (Southwick, 1993; Mellman et al., 1995c; Geracioti, 2001; O’Donnell et al., 2004; Harvey et al., 2006; Adamec et al., 2007; Olson et al., 2011; George et al., 2013). The increased sympathetic nervous system activation (Task Force of the European Society of Cardiology and the North American Society of Pacing and Electrophysiology, 1996), as well as pharmacological interventions (Pitman et al., 2002; Vaiva et al., 2003; Taylor et al., 2006; Raskind et al., 2007; Stein et al., 2007; Hoge et al., 2012) also point toward elevated NA activity during REMS in PTSD patients (Mellman et al., 2004; Woodward et al., 2009), and REMS continuity is interrupted by phasic increases in NA tone under physiological conditions (Fort et al., 2009; Luppi et al., 2012). It remains to be shown in future studies in mice, whether NA hyperactivity, indeed, contributes to reduced REMS continuity and development of hyperarousal.

Our study holds some limitations, which have to be considered while interpreting our findings. First, we did not perform any behavioral testing directly or 2 months after the shock, in order to avoid confounding influences on EEG recordings, as seen in non-shocked mice right after control exposure to the shock context. Consequently, we cannot be entirely sure that PTSD-like symptoms were maintained until the end of the recordings (i.e., day 55). This, however, is rather likely, even if mice were re-exposed to the shock context for 3 min at day 28 (Golub et al., 2009; Siegmund et al., 2009b), also since this protocol did not result in extinction of PTSD-like symptoms in previous studies (Thoeringer and Wotjak, unpublished observations). Accordingly, REMS changes observed at day 1 correlated significantly with those measured at day 55 (*r*^2^ = 0.54, *p* < 0.05), thus demonstrating the persistence of REMS changes despite behavioral testing 1 month after trauma.

Second, we are aware of the fact that correlations do not measure causality and that the low sample size of *n* = 8 limit the conclusions drawn from this study. In light of the resulting low power, we decided to not correct the significance level for multiple correlation analyses. However, we obtained strong correlations independently of whether parametric (Pearson) or non-parametric (Spearman) procedures were employed.

Third, it is a general shortcoming of animal models of psychiatric disorders that it is difficult to judge whether a behavioral phenotype is adaptive or maladaptive. Such categories require an environmental context (does the behavioral change hinder the survival in a distinct environment?) and an evolutionary perspective (does the behavioral change hinder the reproduction success?). In addition, as long as we do not explicitly interfere with the changes in REMS, we can only speculate about their maladaptive nature. An increase in freezing *per se* (and coinciding changes in REMS) should be interpreted with caution. However, concerning the large amount of studies reporting REMS changes and hyperarousal in PTSD patients, we see clear face validity to the human disease and interpret the behavioral and electrophysiological data obtained in the present study in terms of disease-like alterations.

Fourth, in human being the prevalence rate for developing PTSD ranges between 8 and 20% of those confronted with a trauma. Also in our mouse model of PTSD, “responders” and “non-responders” can be observed (Siegmund and Wotjak, 2007a; Siegmund et al., 2009b), depending – among others – on maternal inexperience (Siegmund et al., 2009a). The prevalence rate in the model though is higher than in humans, probably due to the choice of a high susceptible strain (Siegmund and Wotjak, 2007a; Dahlhoff et al., 2010). The broad variance shown by the animals in both freezing and acoustic startle (see correlation analyses) support the existence of high- vs. low-susceptible animals also in case of the present study. However, the sample size is too small to allow for a direct comparison of “high responders” vs. “low responders.”

In summary, our data indicate that low baseline REMS continuity could predict the individual susceptibility for developing PTSD-like hyperarousal symptoms. Fragmented REMS has been associated with the development of PTSD symptoms in humans (Mellman et al., 2002; Breslau et al., 2004; van Liempt et al., 2013) and early life trauma-related REMS fragmentation has been suggested to increase the vulnerability to psychopathologies in adulthood (Insana et al., 2012). Therefore, especially in occupational fields with daily experience of traumatic situations, such as emergency assistance and combat missions, REMS architecture might constitute a viable, objective and non-invasive risk marker for the development of PTSD, and other trauma-related psychiatric disorders before trauma.

## Conflict of Interest Statement

The authors declare that the research was conducted in the absence of any commercial or financial relationships that could be construed as a potential conflict of interest.
